# HEALTH PROMOTION TO PREVENT AND REDUCE COGNITIVE DECLINE IN LATINOS LIVING WITH HIV/AIDS

**DOI:** 10.1093/geroni/igad104.1683

**Published:** 2023-12-21

**Authors:** Elliott Weinstein, Marcela Kitaigorodsky, Victoria Behar-Zusman, Daniel Jimenez

**Affiliations:** University of Miami, Miami, Florida, United States; Florida Neuro-Health, Hollywood, Florida, United States; University of Miami, Miami, Florida, United States; University of Miami Miller School of Medicine, Miami, Florida, United States

## Abstract

Older Latinos living with HIV (LWH) are at increased risk for earlier onset of aging-related cognitive decline. HIV-related cognitive impairments are observed in several domains including memory, reasoning/executive functioning, and speed of processing. Depression, a known neurocognitive risk, has been reported at five times the level among older Latinos LWH than in the general population, and older Latinos LWH are more likely to be sedentary, and not as actively engaged in pursuing changes in physical activity compared to their non-Latino White counterparts. Thus, this is a population that is particularly vulnerable to cognitive decline due to multiple risk factors. The aim of this study is to assess feasibility, acceptability, and preliminary intervention effects on cognition of the Happy Older Latinos are Active (HOLA) health promotion intervention. Thirty Latinos living with HIV with a mean age of 61.6 years (SD=6.1) were enrolled in a pilot single-arm trial. Participants were assessed at two time points on measures of cognitive and psychosocial functioning as well as biomarkers of cognition. In 7 months, we met our enrollment target with <5% of eligible participants refusing participation. Participants attended over 70% of sessions and 3 participants were lost to follow up. These results indicate that HOLA is an innovative health promotion program that is uniquely tailored to address the multiple concerns that are prevalent in this community in a non-stigmatizing and culturally acceptable manner.

